# FoxO1 regulates TLR4/MyD88/MD2‐NF‐κB inflammatory signalling in mucosal barrier injury of inflammatory bowel disease

**DOI:** 10.1111/jcmm.15075

**Published:** 2020-02-14

**Authors:** Chenyang Han, Li Guo, Yongjia Sheng, Yi Yang, Jin Wang, Yanling Gu, Wenyan Li, Xiaohong Zhou, Qingcai Jiao

**Affiliations:** ^1^ State Key Laboratory of Pharmaceutical Biotechnology School of Life Science Nanjing University Nanjing China; ^2^ Department of pharmacy The Second Affiliated Hospital of Jiaxing University Jiaxing China; ^3^ Department of Center Laboratory The Second Affiliated Hospital of Jiaxing University Jiaxing China; ^4^ Department of gastroenterology The Second Affiliated Hospital of Jiaxing University Jiaxing China

**Keywords:** inflammatory bowel disease, inflammatory signal, tight junction, transcription factor FoxO1

## Abstract

In this study, FoxO1 transgenic mice (transgenic, FoxO1‐Tg) and C57BL/6 wild‐type (wild‐type, FoxO1‐WT) mice were used to establish chronic colitis by drinking water containing dextran sulphate sodium (DSS). Afterwards, we observed the life changes in mice and assessed the pathological changes by H&E tissue staining. In addition, the TLR4/MyD88/MD2‐NF‐κB inflammatory signals were detected. As a result, under DSS treatment, the activation level of TLR4/MyD88/MD2‐NF‐κB inflammatory signal was higher in FoxO1‐Tg mice than that in FoxO1‐WT mice. Meanwhile, the intestinal mucosal tissue damage was more severe, the down‐regulation of tight junction protein level was more significant and the life quality was decreased to a higher degree in FoxO1‐Tg mice compared with those in FoxO1‐WT mice. Caco‐2 cells were used to mimic the intestinal mucosal barrier model for in vitro assays. In addition, lentiviral packaging FoxO1 overexpressing plasmid was transfected into Caco‐2 cells for FoxO1 overexpression. TNF‐α intervention was performed for intestinal mucosal inflammatory response model. Consequently, the down‐regulation of FoxO1 inhibited the activation of TLR4/MyD88/MD2‐NF‐κB inflammatory signal, decreased the mucosal barrier permeability and up‐regulated the expression of tight junction protein. By contrast, the overexpression of FoxO1 increased the mucosal barrier permeability and down‐regulated the level of tight junction protein.

## 1 INTRODUCTION

Inflammatory bowel disease (IBD) is a type of inflammatory disease that occurs in the digestive tract, with chronic and recurrent course, which can be mainly divided into Crohn's disease (CD) and ulcerative colitis (UC). In CD, inflammation is usually distributed throughout the entire digestive tract, characterized by segmental lesion. Epidemiological studies have shown that IBD is prevalent in people aged 20‐40 years.^1^, ^2^ The intestinal mucosal epithelial cells are mainly composed of goblet cells, Paneth cells, M cells and absorptive cells, with villus structure. The occurrence of IBD mainly damages the local mucosal structure, which is characterized by the loss of tight junction proteins, the damage of intestinal epithelial cells and the destruction of villus structure, among which, local inflammatory response plays an important role.^3^, ^4^ Toll‐like receptors (TLRs) are the main receptors that mediate inflammatory responses. After stimulation by HMGB1, LPS,etc, TLRs can further transmit signals to activate downstream NF‐κB, thereby promoting the expression of inflammatory factors such as interleukin (IL) and tumour necrosis factor (TNF) and interferon (IFN). Among them, TLR4 is a relatively critical receptor, and TLR4, MD2 and MyD88 combine to form a complex,^5^ playing an important role in the early stage of inflammatory signals. However, the regulation mechanism of this signal in IBD has not been clarified at present.

Transcription factors (TFs) are a group of proteins that regulate the transcription process and regulate protein expression through transcriptional and translational modulation of mRNA, further regulating protein functions.^6^, ^7^ A variety of TFs have been found to be involved in UC. Our team has found that the expression of FoxO1, a transcription factor, is increased in the intestinal tissue of patients with IBD. FoxO1, a member of the Fox family, is involved in apoptosis, stress, DNA damage, metabolism and etc Moreover, FoxO1 has been found to promote the carcinogenesis and tumour progression in a variety of tumours. However, to date, the role of FoxO1 has not been reported in IBD. The expression of FoxO1 and TLR4 is high in IBD. Previous studies have also reported the association between TLR4 and FoxO1 in anti‐inflammatory response.^8^ However, whether there is a regulatory relationship between them has not been reported. As IBD is a risk factor of colorectal cancer, we cautiously speculate that FoxO1 might play a role in the transformation from colitis to colorectal cancer. Herein, in this study, we mainly investigated the role of FoxO1 in IBD.

## 2 MATERIALS AND METHODS

### 2.1 Detection of the expression of FoxO1 protein in tissues of IBD patients

A total of 30 patients with IBD admitted to the Department of Gastroenterology at the Hospital from **June 2017 to May 2018**. This included 15 patients with CD and 15 patients with UC. In addition, another 15 healthy people who underwent physical examination during the same period were collected. 30 patients were composed 18 males and 12 females. Their age was 39‐65, Inclusion criteria: (a) Clinical manifestations: The patient has persistent or recurrent diarrhoea, mucopurulent stool and abdominal pain. (b) Endoscopy: Patients with intestinal lesions from the beginning of the rectum and showed a continuous, diffuse distribution. (c) Pathological examination: The inflammatory reaction and pathological changes in intestinal tissue were observed by naked eye and histology.

All patients underwent colonoscopy, followed by acquisition of colon tissue for biopsy. All patients signed the written informed consent. Intestinal protein from patients was extracted to detect the protein expression of FoxO1 by ELISA kit. Moreover, paraffin‐embedded tissue was subjected to immunohistochemical staining to detect the expression of FoxO1.

The study was approved by the Ethics Committee of The Second Affiliated Hospital of Jiaxing University (Chenyang Han,Li Guo,Yongjia Sheng,Yi Yang,Jin Wang,Yanling Gu,Wenyan Li,Xiaohong Zhou belongs to The Second Affiliated Hospital of Jiaxing University). Animal experiments conform to the ethical norms of animal experiments and the relevant provisions of animal welfare. All patients or their family members signed written informed consent and follow‐up consent at the time of initial diagnosis.

### 2.2 Establishment and grouping of animal models

Transgenic mice (FMS‐FoxO1‐GFP‐transgenic, Tg mice, Saiye Biotechnology Co., Ltd.) with high expression of FoxO1 and wild‐type C57BL/6 mice (wild‐type, WT mice) were fed under the same environment. After adaptive feeding for one week, WT and Tg mice were randomly divided into FoxO1‐WT, FoxO1‐Tg, FoxO1‐WT‐DSS and FoxO1‐Tg‐DSS groups (N = 5 in each group). Chronic colitis model was constructed by daily feeding of 2.5% DSS in drinking water at 1‐5, 8‐12, 15‐19 and 22‐26 days, whereas distilled water was supplied for the rest of the time, which lasted for a total of four cycles. DAI scores and mouse weight loss rate were measured on days 1, 3 and 5 of each cycle. Mice were killed on day 29 by carbon dioxide asphyxiation. Afterwards, mice were placed in the supine position, disinfected with iodine, and the abdominal skin and muscle layer were quickly cut off to separate the terminal ileum until to the rectum. The resected tissue was washed with PBS for three times, fixed with 4% paraformaldehyde and embedded with paraffin for further analysis.

### 2.3 Evaluation of bodyweight and DAI score in mice with chronic colitis

The DAI score was used to evaluate the pathological state of mice. In addition, the mental state, activity, hair gloss, appetite, faecal traits and bodyweight of mice were observed daily. According to the criteria of DAI score (shown in Table 1), the bodyweight, faecal traits and occult blood of mice were assessed and recorded on days 1, 3 and 5 of each cycle. In terms of bodyweight loss rate, bodyweight of mice was measured on days 1, 3 and 5 of each cycle, followed by calculation of ratio between the bodyweight and initial bodyweight. After the mice were killed, the colon length was determined (shown in cm). Moreover, the gross morphological score of colonic injury in mice was performed according to the relevant criteria (shown in Table 2).

**Table 1:** DAI score criteria

Score	Bodyweight loss (%)	Faecal traits	FOB
0	<1	Less in amount, stiff, dry, not sticky, solid	No colour
1	1‐5	Less in amount, stiff, wet, sticky	Spotted colour
2	6‐10	More in amount, soft, very sticky	Blue
3	11‐15	More in amount, soft, scattered	Dark stool+ blue FOB
4	>15	Loose stools	Bloody stool+blue FOB

Note

DAI=percentage of bodyweight loss＋faecal trait score＋FOB score

**Table 2:** Scoring criteria of gross morphology of colon

Gross morphology	Score
Normal gross morphology	0
Slightly thickened of colon wall but not congested	1
Moderately thickened of colon wall with congestion	2
Significantly thickened and stiff of colon wall with congestion	3
Significantly thickened and stiff of colon wall with congestion and adhesion	4

### 2.4 Histopathological examination of mouse intestinal tissue (H&E staining)

Paraffin‐embedded colon tissue was serially cut into 4‐μm‐thick sections, dewaxed with xylene, dehydrated with gradient concentrations of 100%, 95%, 80% ethanol, rinsed with tap water for 2 minutes, stained with haematoxylin for 3 minutes, rinsed with tap water for 2 minutes, treated with 1% hydrochloric acid ethanol for 2 seconds, rinsed with tap water for 2 minutes, reacted with 1% ammonia for 20 seconds, stained with 0.5% eosin ethanol for 10 seconds, dehydrated with gradient ethanol, treated with xylene for transplantation and sealed with neutral gum. Finally, the pathological changes in intestinal tissue were observed under light microscope.

### 2.5 Ultrastructural changes in colon tissue by transmission electron microscope

The colon tissue was placed in 2.5% pre‐cooled glutaraldehyde fixative (imported specific for electron microscope). The sample was cut into a volume of 1 mm^3^, transferred to a 1.5 mL EP tube and fixed at 4°C overnight. Pre‐fixation: 4°C, fixed for 24 hours, rinsed with 0.1 mol/L PBS 3 times (10 minutes each). Post‐fixation: 1% citric acid, fixed at 4°C for 2 hours, rinsed with 0.1 mol/L PBS 3 times (10 minutes each). Dehydration: 30%, 50%, 70%, 80%, 95%, 100% acetone (15 minutes each). Soaking: (a) acetone, Epon812 resin (V = 3:1), 2 hours; (b) acetone, Epon812 resin (V = 1:1), 3 hours; (c) acetone, Epon812 resin (V = 1:3), 3 hours; (d) Epon812 resin for 6 hours. Embedding: Epon812 resin 37°C, overnight; 45°C, 12 hours; 60°C, 24 hours. Staining: Samples were cut into ultra‐thin sections (60 nm) by a cryostat. Staining and photography: Sections were stained with 1% acetate shaft staining for 20 minutes and stained with lead citrate for 7 minutes, followed by observation and photography under transmission electron microscope (TEM).

### 2.6 Detection of tight junction protein expression by immunohistochemistry (IHC)

The paraffin‐embedded intestinal tissue samples were cut into 4‐μm‐thick sections, baked at 60°C for 2 hours, dewaxed in xylene 3 times (5 minutes each), immersed in absolute ethanol for 5 minutes, immersed with 95% ethanol 2 times (2 minutes each), immersed in 85% ethanol once for 2 minutes; rinsed with tap water for 5 minutes and rinsed with distilled water for 3 minutes. Afterwards, antigen retrieval was performed in 0.01 mol/L citrate buffer (pH = 6.0) by microwave at 98°C for 20 minutes, followed by cooling down at room temperature for 30 minutes and then rinsing with distilled water. The sections were incubated with 3% hydrogen peroxide to eliminate endogenous peroxidase at room temperature for 10 minutes. Afterwards, sections were blocked with 2% bovine serum albumin (BSA) at 37°C for 30 minutes to prevent the non‐specific binding between antigen and antibody. After discarding BSA, the sections were incubated with proper primary antibody at 37°C for 2 hours. The primary antibodies included ZO‐1 and occludin (dilution 1:300 and 1:500, respectively, Abcam). After washing with TBS 3 times (5 minutes each), the sections were reacted with corresponding secondary antibody at 37°C for 15 minutes, followed by incubation with peroxidase‐labelled streptomycin (Abcam) for 15 minutes. After rinsing with PBS for 3 times (5 minutes each), each section was dropped with freshly prepared DAB solution (DAKO), and the reaction was terminated by observing under microscope. Subsequently, the sections were rinsed with tap water, counterstained with haematoxylin and sealed. For negative control, primary antibody was replaced by TBS. All sections were photographed under Olympus‐BX51 upright microscope with Olympus‐DP72 image acquisition system and CRi Nuance multispectral imaging system (Cambridge Research & Instrumentation).

### 2.7 Detection of colonic mucosal permeability by FITC‐D

After relevant intervention, mice were fasted and free of water 4 hours before killing. Fluorescein isothiocyanate‐dextran (SM Biochemicals, FITC‐D, MW 4000) was administered orally (60 mg FITC‐D /100 g), followed by collection of serum. The fluorescence density of each sample was measured by fluorescence spectrophotometer, and the serum concentration of FITC‐D was examined as well.

### 2.8 Relative expression levels of proteins by Western blot

In total, 100 mg of colon tissue was cut into pieces using sterile surgical scissors. For cell assay, cells were collected, washed with PBS for 2 times and centrifuged for collection. Both tissues and cells were ground within liquid nitrogen, followed by addition of 1.0 mL of RIPA lysate (Biyuntian Biotechnology Co., Ltd.) on ice for 30 minutes. After centrifuging at 10 000 *g* for 15 minutes, the supernatant was subjected to protein quantification. Meanwhile, SDS‐PAGE was prepared. Protein sample was mixed with 5 × loading buffer, boiled for 8 minutes, subjected to SDS‐PAGE at 80 V and then 120 V, and transferred to PVDF membrane at 300 mA for 0.5‐2 hours. The PVDF membranes were blocked with 5% skim milk for 2 hours and incubated with primary antibodies in TBST. Afterwards, the membranes were washed with TBST twice and incubated with horseradish peroxidase‐labelled goat anti‐rabbit secondary antibody (dilution 1:20000, Abcam). Afterwards, ECL method was used for visualization, followed by analysis of the optical density using Image Pro‐Plus 6.0 software. GAPDH was used as the internal control. The results were shown as comparison of optical density values between the target protein and the internal control protein. Dilution ratio of monoclonal antibody: FoxO1, ZO‐1, occludin (CST, Dilution 1:200), TLR4, MyD88, IkB, p‐p65, MD2 (Abcam, Dilution 1:100‐1:300).

### 2.9 Construction of cell lines with FoxO1 overexpression/down‐regulation

Small interfering RNA (siRNA) was used to silence FoxO1 expression. In brief, cells were cultured in DMEM medium (Gibco) with 100 ml/L FBS, and cells in the logarithmic phase were used for further analysis. During transfection, Opti‐MEM medium (Gibco) was used. In addition, 200 pmol of siRNA (Genepharm Biotechnology Co., Ltd.) and 5 μL of Lipofectamine 2000 were separately diluted with 250 μL of Opti‐MEM and incubated at room temperature for 5 minutes. Afterwards, 250 μL of Lipofectamine 2000 was mixed with 250 μL of siRNA to obtain a total volume of 500 μL and incubated at room temperature for 20 minutes. The mixture was separately added to a 6‐well plate, mixed and cultured for 6 hours. Afterwards, Opti‐MEM was discarded and fresh complete medium was added for additional incubation of 48 hours. For constructing overexpression cell line, lentiviral packaging FoxO1 overexpressing plasmid was transfected using the similar transfection protocol.

### 2.10 Determination of transepithelial electrical resistance (TEER)

The cells were seeded in Transwell chamber at a density of 4 × 10^5^ cells/per well (200 μL per well). After inoculation for 24 hours, the medium was changed, which was thereafter changed every other day to monitor TEER. TEER was measured by Millicell resistance meter. Briefly, the two electrodes of the resistance meter were inserted into the top side and basal side of the chamber and immersed within the liquid to measure TERR. The assay was conducted at 37°C, and three points from different directions of each Transwell chamber were taken, and the assay was conducted in triplicate. The resistance value was expressed as ohm/cm^2^ (Ω/cm^2^). In consideration of the intrinsic electrical resistance of the Transwell membrane, the standard TEER value = (measured value − blank control value)/0.33 cm^2^.

### 2.11 FITC‐D permeability measurement

FITC‐D was used as a marker to examine the effect of different interventions on paracellular shunt in Caco‐2 cells. The cells were carefully rinsed with Hank's Balanced Salt Solution (HBSS) 3 times, which was further aspirated as much as possible to remove any interfering substances on the cell surface. As for the final wash, cells were incubated in the incubator for 30 minutes, followed by aspiration of HBSS. Afterwards, FITC‐D was added to the top of the Transwell chamber at a final concentration of 1 mg/mL, and 0.6 mL of HBSS was added to the basal side. After incubation at 37°C for 1 hours, the liquid of the basal side was collected, followed by determination of the fluorescence intensity by fluorescence spectrophotometer (excitation wavelength 490 nm and emission wavelength 520 nm). The concentration of FITC‐D was calculated according to the standard curve of FITC‐D. FITC‐D permeability (%/h/cm^2^) = (basal side FITC‐D fluorescence intensity/ initially added FITC‐D fluorescence intensity)/1 hours/0.33 cm^2^ × 100%.

### 2.12 Statistical analysis

Measurement data were shown as mean ± standard deviation ($\bar{\chi }$ ± s). One‐way ANOVA was used for comparison among multiple groups, and LSD as post hoc, SNK test was utilized for comparison between groups. SPSS 18.0 software was used for statistical analysis. A *P* < .05 was considered as statistical significance.

## 3 RESULTS

### 3.1 Expression of FoxO1 in intestinal tissues of IBD patients

**Figure 1:**
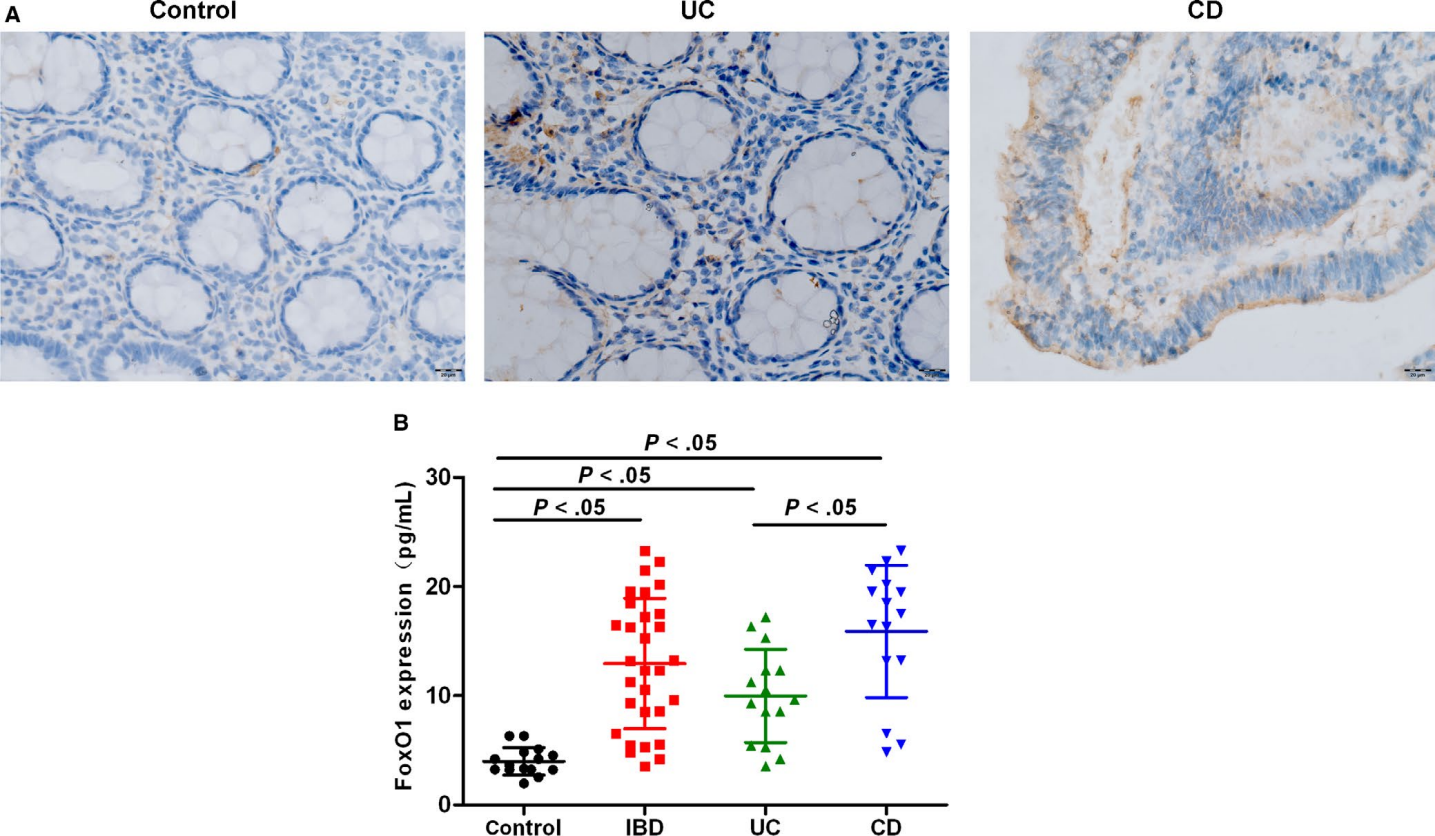
Expression levels of FoxO1 in intestinal tissues of IBD patients (Control: healthy population; IBD: inflammatory bowel disease; UC: ulcerative colitis; and CD: Crohn's disease). A, IHC was used to detect the expression of FoxO1 in intestinal tissues of IBD patients (brown for positive FoxO1 protein staining). As a result, the protein level of FoxO1 was higher in UC and CD patients than Control. B, ELISA was used to determine the protein level of FoxO1 in intestinal tissue of IBD patients (UC+CD). Comparison between groups, *P*<.05

Immunohistochemistry showed the low expression of FoxO1 in healthy population (Control), and ELISA revealed that the level of FoxO1 was 4.52 ± 0.87 pg/mL in 15 healthy people. However, FoxO1 was significantly up‐regulated in intestinal tissue of IBD patients. Additionally, IHC showed that the expression of FoxO1 was significantly higher in CD group than that in UC group. ELISA revealed that the level of FoxO1 was 14.32 ± 1.87, 10.85 ± 0.98 and 15.65 ± 2.15 pg/mL in patients with IBD, patients with UC and patients with CD, respectively. Thus, the level of FoxO1 in the intestinal tissue was significantly higher in IBD patients than that of healthy population (shown in Figure 1).

### 3.2 Effects of FoxO1 by evaluating bodyweight, DAI score and pathological condition of mice with chronic colitis

**Figure 2:**
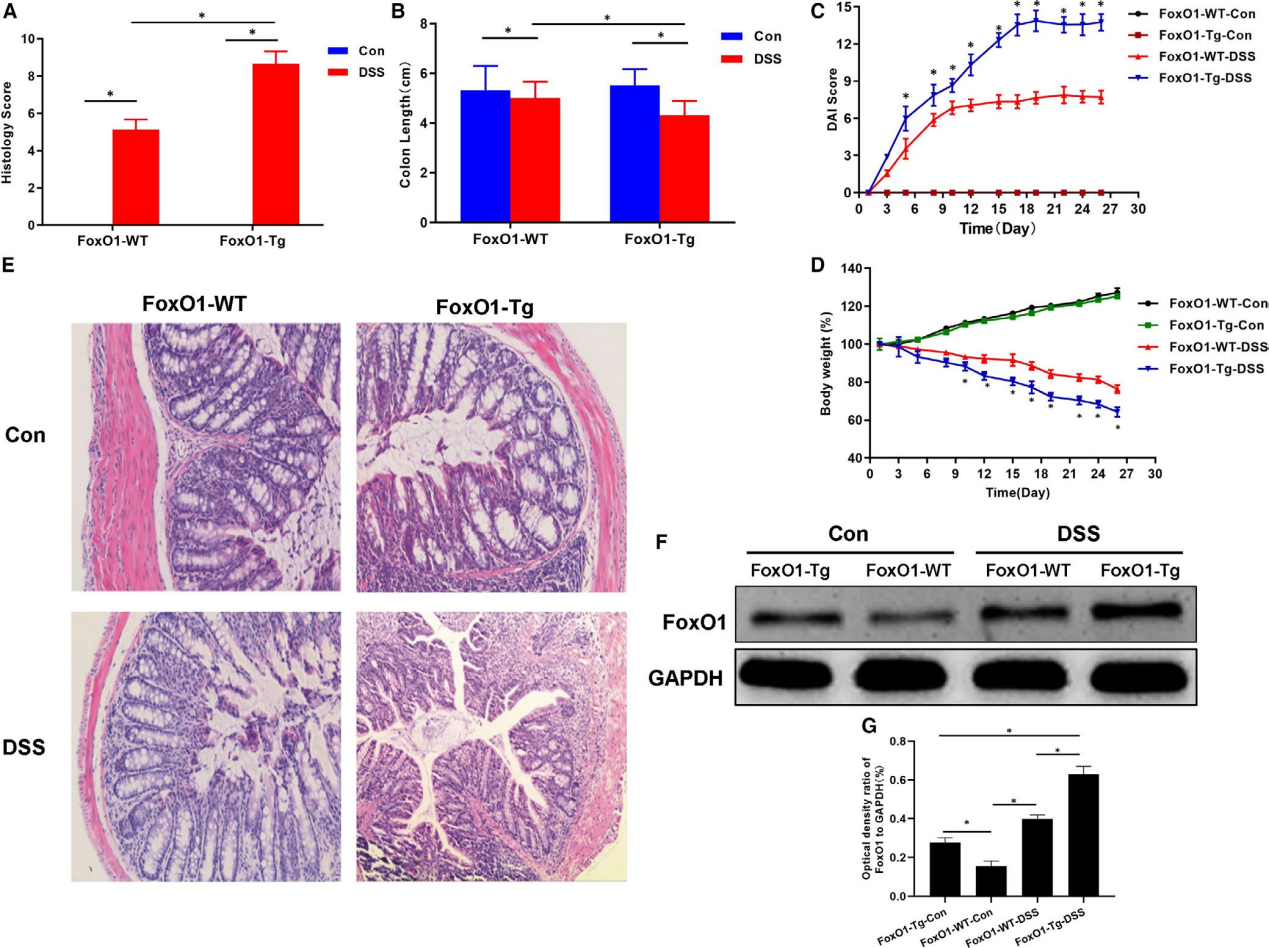
Effects of FoxO1 by evaluating bodyweight, DAI score and pathological condition of mice with chronic colitis. A, The intestinal histopathological scores in mice showed that the pathological scores were 0 in FoxO1‐WT‐Con and FoxO1‐Tg‐Con groups. However, after DSS intervention, the pathological scores were significantly higher in FoxO1‐Tg‐DSS group than those of FoxO1‐WT‐DSS group. Comparison between groups, ^*^ *P*<.05. B, The change in intestinal tissue length in mice: The length of intestinal tissue was significantly lower in FoxO1‐Tg‐DSS group than that in FoxO1‐WT‐DSS group. Comparison between groups, ^*^ *P*<.05. C, DAI scores in mice showed no significant changes in FoxO1‐WT‐Con and FoxO1‐Tg‐Con mice during the experiment, whereas DAI scores were significantly changed in FoxO1‐Tg‐DSS and FoxO1‐WT‐DSS groups. Comparison with FoxO1‐WT‐DSS group at the same time‐point, ^*^ *P*<.05. D, Bodyweight changes of mice: The bodyweight was gradually increased in FoxO1‐WT‐Con and FoxO1‐Tg‐Con mice under normal feeding, without significant difference. However, the bodyweight was decreased in FoxO1‐Tg‐DSS and FoxO1‐WT‐DSS groups. Comparison with the FoxO1‐WT‐DSS group at the same time‐point, ^*^ *P*<.05. E, HE staining showed that the intestinal mucosa epithelium was intact, and the intestinal gland composed of lamina propria and mucosa muscle layer was arranged regularly in FoxO1‐WT‐Con group under light microscope. However, the colonic mucosa was defective, with decreased goblet cells and destructive or even disappeared gland, and large number of lymphocytes were infiltrated in the submucosa and even the muscle layer in FoxO1‐WT‐DSS group, which was more severe in FoxO1‐Tg‐DSS group than FoxO1‐WT‐DSS group. F,G, The expression of FoxO1 in mouse intestinal tissue: The expression of FoxO1 was increased in FoxO1‐WT‐DSS and FoxO1‐Tg‐DSS groups after DSS intervention, and the expression of FoxO1 was significantly higher in FoxO1‐Tg‐DSS group than that in FoxO1‐WT‐DSS group. Comparison between groups, ^*^ *P* < .05

During the experiment, FoxO1‐WT‐Con and FoxO1‐Tg‐Con mice were good in general condition, with normal stool and gradually increased bodyweight. After DSS intervention, the hair was dull, the amount of drinking water was decreased, the frequency of defecation was increased, with the appearance of mucopurulent bloody stool, and the bodyweight was significantly decreased. Moreover, the bodyweight loss was significantly more obvious in the FoxO1‐Tg‐DSS group than that in the FoxO1‐WT‐DSS group. DAI score was not significantly different between FoxO1‐WT‐Con and FoxO1‐Tg‐Con mice, whereas DAI score was significantly increased in FoxO1‐Tg‐DSS and FoxO1‐WT‐DSS mice, and DAI score was significantly higher in FoxO1‐Tg‐DSS mice than FoxO1‐WT‐DSS mice. The HE staining of colonic histopathology showed that the intestinal mucosa epithelium was intact, and the intestinal gland composed of lamina propria and mucosa muscle layer was arranged regularly in FoxO1‐WT‐Con group under light microscope. However, the colonic mucosa was defective, with decreased goblet cells and destructive or even disappeared gland, and large number of lymphocytes were infiltrated in the submucosa and even the muscle layer in FoxO1‐WT‐DSS group, with significantly increased pathological score (5.68 ± 0.52 vs 0.00 ± 0.00, *P* < .05). In the FoxO1‐Tg‐DSS group, there was intestinal mucosal injury in mice, with significantly higher pathological score than that in the FoxO1‐WT‐Con group (11.83 ± 1.05 vs 0.00 ± 0.00, *P* < .05) and the FoxO1‐WT‐DSS group (11. 83 ± 1.05 vs 5.68 ± 0.52, *P* < .05). The expression of FoxO1 was significantly different between FoxO1‐WT‐Con and FoxO1‐Tg‐Con groups. After DSS intervention, the expression of FoxO1 was significantly elevated in FoxO1‐Tg‐DSS and FoxO1‐WT‐DSS groups (shown in Figure 2).

### 3.3 Effects of FoxO1 on the expression of tight junction protein in mice with chronic colitis

**Figure 3:**
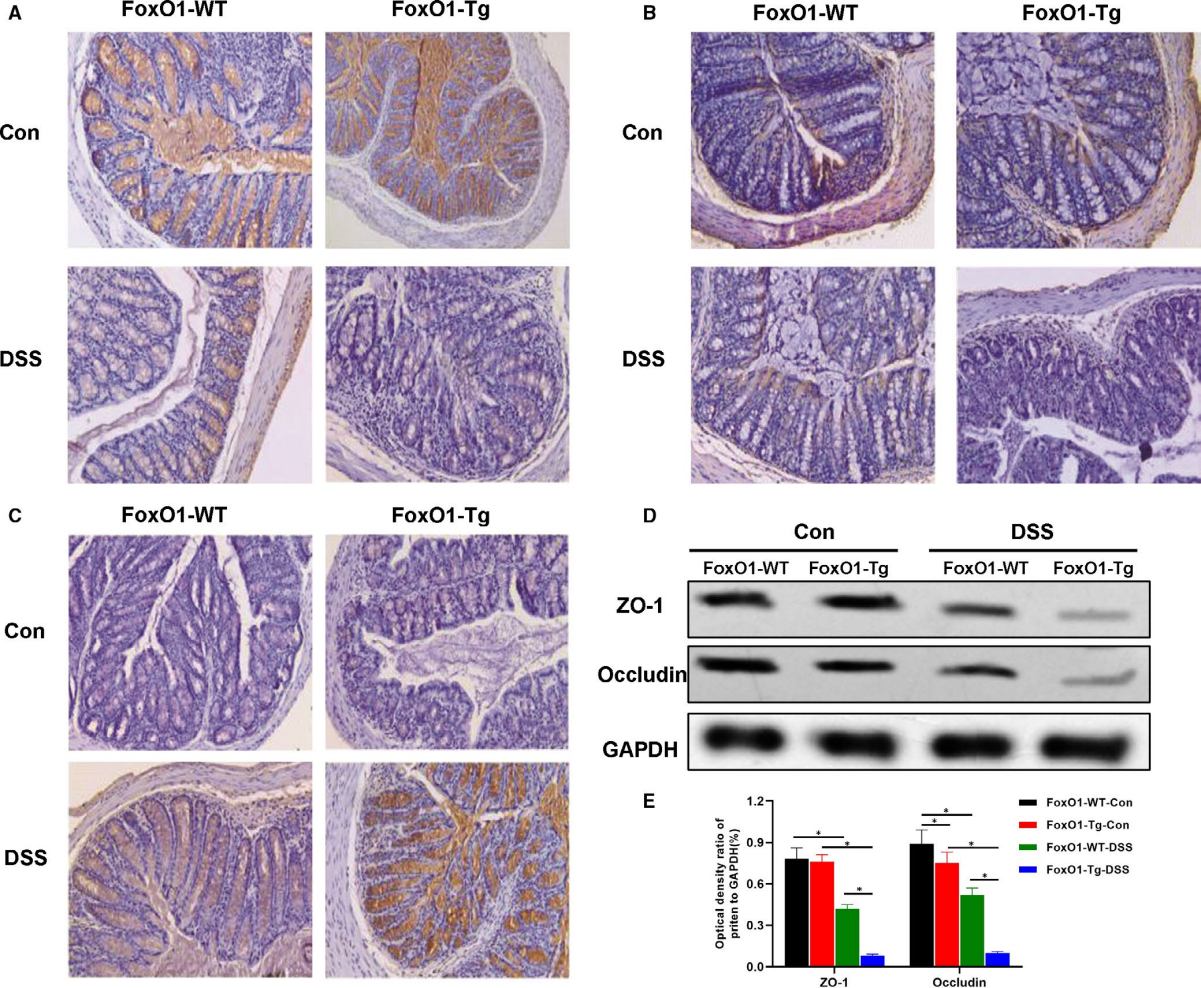
Effects of FoxO1 on the expression of tight junction protein in mice with chronic colitis. A, Expression of ZO‐1 protein in mouse intestinal tissue by IHC: ZO‐1 protein was strongly expressed in intestinal tissues of FoxO1‐WT‐Con and FoxO1‐Tg‐Con groups, which was significantly down‐regulated in FoxO1‐WT‐DSS and FoxO1‐Tg‐DSS groups. Meanwhile, the expression of ZO‐1 was significantly lower in FoxO1‐Tg‐DSS group than that of FoxO1‐WT‐DSS group. B, Expression of occludin protein in mouse intestinal tissue by IHC: Occludin protein was strongly expressed in intestinal tissues of FoxO1‐WT‐Con and FoxO1‐Tg‐Con groups, which was significantly down‐regulated in FoxO1‐WT‐DSS and FoxO1‐Tg‐DSS groups. Meanwhile, the expression of occludin was significantly lower in FoxO1‐Tg‐DSS group than that of FoxO1‐WT‐DSS group. C, Expression of FoxO1 protein in mouse intestinal tissue by IHC: The expression levels of FoxO1 were significantly increased in FoxO1‐WT‐DSS and FoxO1‐Tg‐DSS groups after DSS intervention, whereas the expression levels of FoxO1 were significantly higher in FoxO1‐Tg‐DSS group than those in FoxO1‐WT‐DSS group. D,E, The protein expression of ZO‐1 and occludin in intestinal tissue by Western blot. After DSS intervention, the expression levels of ZO‐1 and occludin were down‐regulated, whereas the expression levels of ZO‐1 and occludin were significantly lower in FoxO1‐Tg‐DSS than those in FoxO1‐WT‐DSS group. Comparison between groups, ^*^ *P*<.05

The expression of tight junction proteins, including ZO‐1 and occludin in the intestinal mucosa of mice was relatively higher in FoxO1‐WT‐Con and FoxO1‐Tg‐Con groups, without significant difference between the two groups (*P* < .05). After DSS intervention and FoxO1 overexpression, the expression of tight junction proteins was down‐regulated, which was more obvious following FoxO1 overexpression. As shown in Figure 3, the expression levels of tight junction proteins, ZO‐1 and occludin were down‐regulated in FoxO1‐WT‐DSS and FoxO1‐Tg‐DSS groups. Consistently, IHC and Western blot both showed the expression levels of tight junction proteins, ZO‐1 and occludin, were significantly decreased in FoxO1‐Tg‐DSS group than those in FoxO1‐WT‐DSS, indicating that DSS intervention caused the down‐regulated expression of tight junction proteins in mouse intestinal mucosa (shown in Figure 3).

### 3.4 Effects of FoxO1 on intestinal mucosal villus structure, permeability and expression of inflammatory signals in mice with chronic colitis

**Figure 4:**
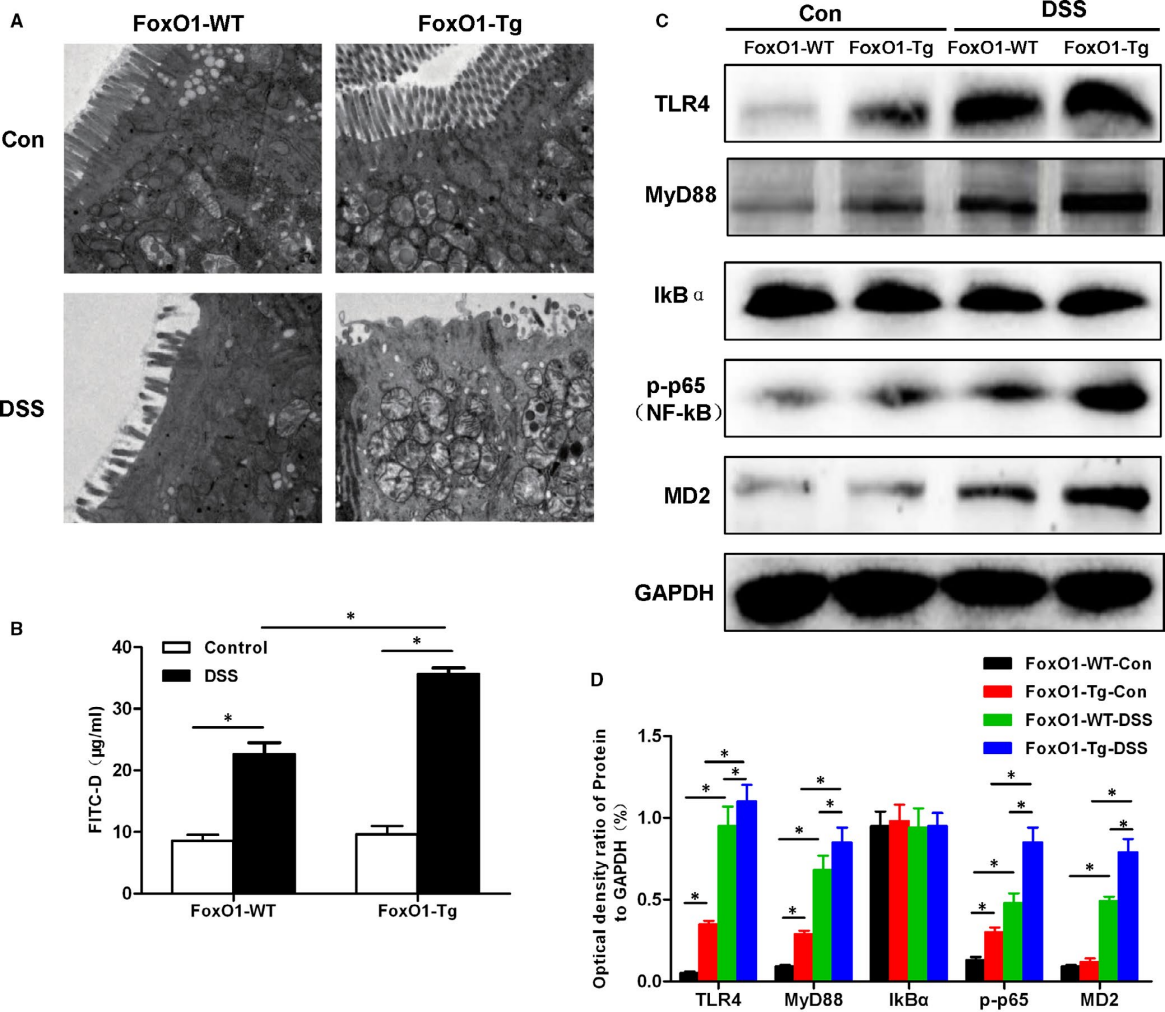
Effects of FoxO1 on intestinal mucosal villus structure, permeability and expression of inflammatory signals in mice with chronic colitis. A, Changes in the intestinal mucosal microvillus structure of mice. The intestinal mucosal microvillus was neatly arranged in FoxO1‐WT‐Con and FoxO1‐Tg‐Con mice, with clear structure. The intestinal mucosa microvillus structure was significantly changed in FoxO1‐WT‐DSS and FoxO1‐Tg‐DSS groups, with obvious loss of microvillus structure. In addition, the lesion degree was significantly more severe in FoxO1‐Tg‐DSS group than that in FoxO1‐WT‐DSS group. B, FITC‐D permeability assay revealed that the permeability was significantly higher in FoxO1‐WT‐DSS and FoxO1‐Tg‐DSS groups than that in Con group. Comparison between groups ^*^ *P*<.05. C,D, Western blot assay for TIL4/MyD88/MD2‐NF‐kB signalling. The activation levels of TIL4/MyD88/MD2‐NF‐kB signalling were lower in FoxO1‐WT‐Con and FoxO1‐Tg‐Con groups. After DSS intervention, the levels of key proteins, TLR4, MyD88 and MD2 were up‐regulated. Comparison between groups, ^*^ *P*<.05

Under TEM, the intestinal mucosal villus structure was neatly arranged in FoxO1‐WT‐Con and FoxO1‐Tg‐Con mice, with clear structure and no change in the villus. After DSS intervention, the intestinal mucosa villus structure was significantly changed in FoxO1‐WT‐DSS and FoxO1‐Tg‐DSS groups, with obvious loss of villus structure. In addition, the degree of lesion was significantly more severe in FoxO1‐Tg‐DSS group than that in FoxO1‐WT‐DSS group. FITC‐D permeability assay showed that the permeable level of FITC‐D was increased after DSS intervention, which was significantly higher in FoxO1‐Tg‐DSS group than that in FoxO1‐WT‐DSS group. Western blot assay showed that TIL4/MyD88/MD2‐NF‐kB signal was significantly activated after DSS intervention, with significantly up‐regulated p‐p65 level and other key proteins, including TLR4, MyD88 and MD2. Moreover, after FoxO1 overexpression, the signal activation was significantly higher than WT (shown in Figure 4.)

### 3.5 Cell permeability changes in inflammatory models after down‐regulation/overexpression of FoxO1 in Caco‐2 cells

**Figure 5:**
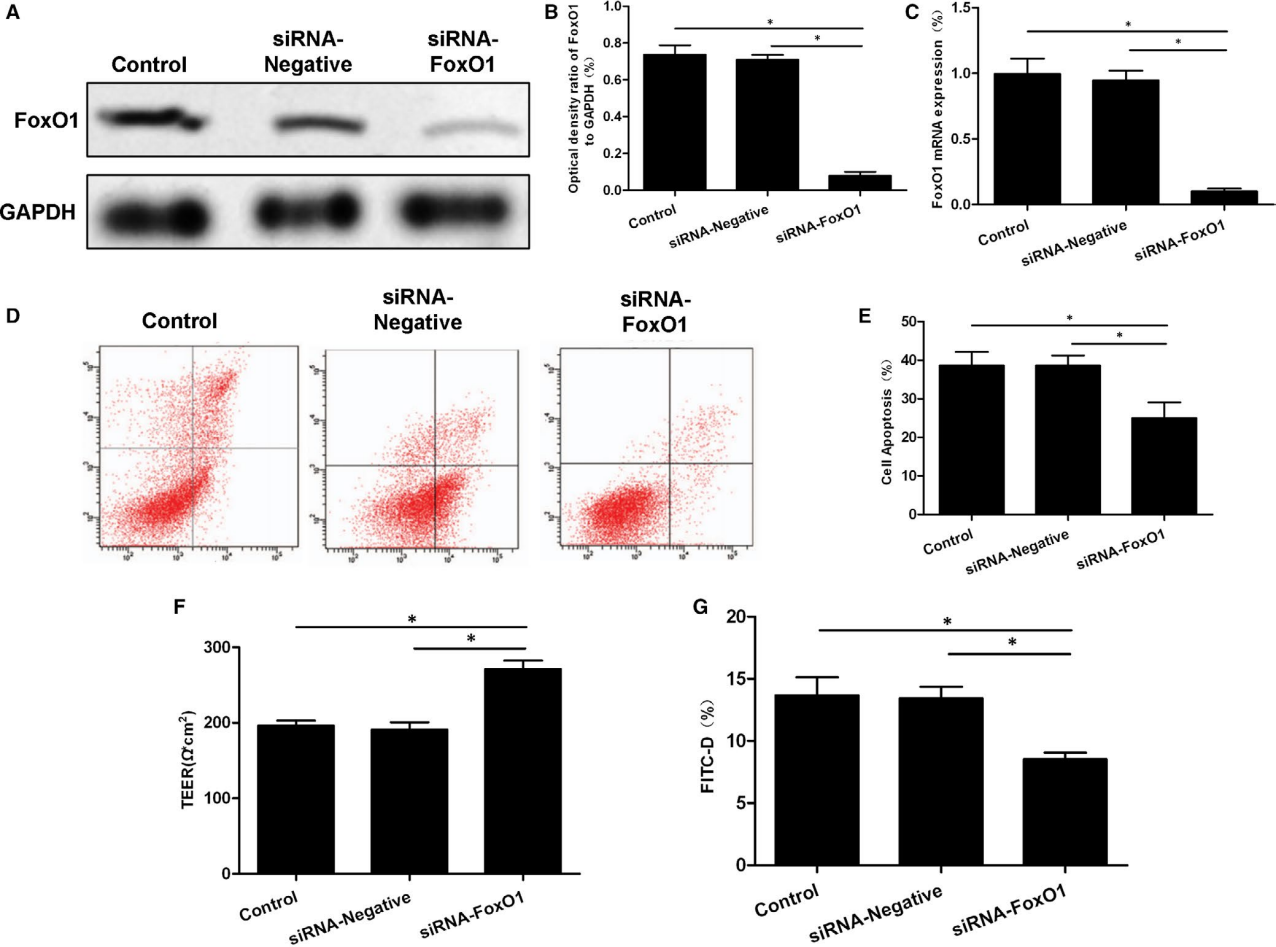
Effects of FoxO1 silencing by siRNA on Caco‐2 cell permeability. A‐C, The protein and mRNA expression of FoxO1 after FoxO1 silencing. After FoxO1 silencing by siRNA, both protein and mRNA levels of FoxO1 were significantly down‐regulated. Comparison between groups, ^*^ *P*<.05. D,E, After TNF‐α intervention, the apoptotic level was not significantly different between the Control group and the siRNA‐Negative group, whereas the apoptotic rate was significantly decreased in the siRNA‐FoxO1 group. Comparison between groups, ^*^ *P*<.05. F, Cell resistance assay showed no significant difference between the Control group and the siRNA‐Negative group, whereas cell resistance was significantly up‐regulated in the siRNA‐FoxO1 group. Comparison between groups, ^*^ *P*<.05. G, The permeability change of FITC‐D was not significantly different between the Control group and the siRNA‐Negative group, whereas the permeability was significantly down‐regulated in the siRNA‐FoxO1 group. Comparison between groups, ^*^ *P*<.05

After silencing FoxO1 by siRNA (the protein level of FoxO1 was down‐regulated), cells were divided into Control, siRNA‐Negative and siRNA‐FoxO1 groups. TNF‐α (TNF‐α intervention with final concentration of 20 μM) was used to construct the inflammatory model in three groups of cells. As a result, apoptosis was obvious, cell permeability was increased and the resistance was decreased in Caco‐2 cells, which were not significantly different between the Control group and the siRNA‐Negative group. However, the cell permeability, resistance down‐regulation and apoptotic rate of the siRNA‐FoxO1 group were significantly different from those of the other two groups. Down‐regulation of FoxO1 could significantly inhibit cell permeability and resistance (shown in Figure 5).

**Figure 6:**
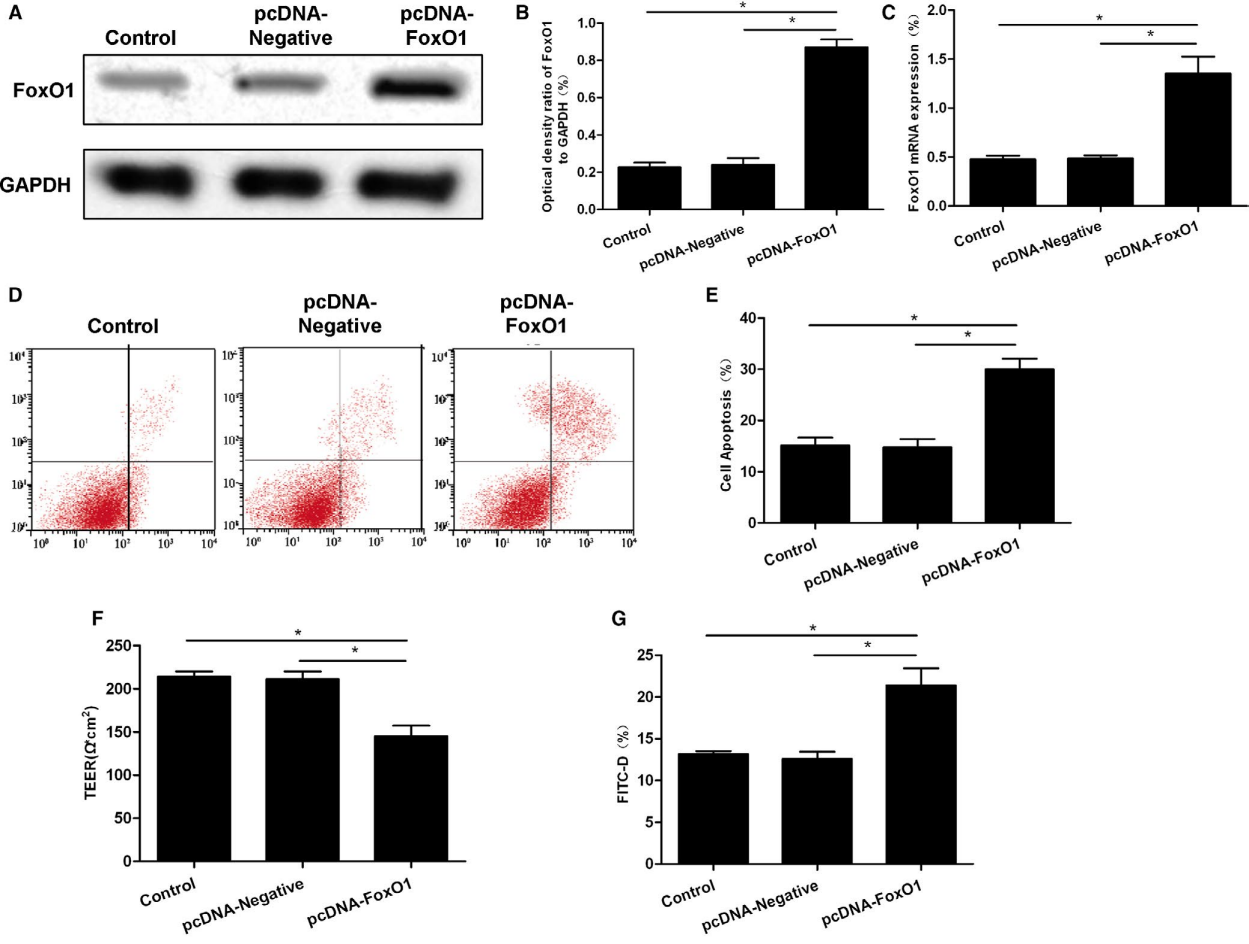
Effects of FoxO1 overexpression on Caco‐2 cell permeability. A‐C, The protein and mRNA expression of FoxO1 after overexpression of FoxO1. After overexpression of FoxO1, both protein and mRNA levels of FoxO1 were significantly up‐regulated. Comparison between groups, ^*^ *P*<.05. D,E, After TNF‐α intervention, the apoptotic level was not significantly different between the Control group and the siRNA‐Negative group, whereas the apoptotic rate was significantly increased in the siRNA‐FoxO1 group. Comparison between groups, ^*^ *P*<.05. F, Cell resistance assay showed no significant difference between the Control group and the siRNA‐Negative group, whereas cell resistance was significantly down‐regulated in the siRNA‐FoxO1 group. Comparison between groups, ^*^ *P*<.05. G, The permeability change of FITC‐D was not significantly different between the Control group and the siRNA‐Negative group, whereas the permeability was significantly up‐regulated in the siRNA‐FoxO1 group. Comparison between groups,^ *^ *P*<.05

The lentiviral packaging pcDNA‐FoxO1 plasmid was transfected into Caco‐2 cells to significantly elevate FoxO1 expression, and cells were further divided into Control, pcDNA‐Negative and pcDNA‐FoxO1 groups. Inflammatory model was constructed by TNF‐α, which caused significant apoptosis. The apoptotic rate was significantly up‐regulated, cell permeability was increased, whereas resistance was down‐regulated in pcDNA‐FoxO1 cells, compared with those in Control and pcDNA‐Negative groups (shown in Figure 6).

### 3.6 Down‐regulation/overexpression of FoxO1 on inflammatory signalling and tight junction protein expression in inflammatory models in Caco‐2 cells

**Figure 7:**
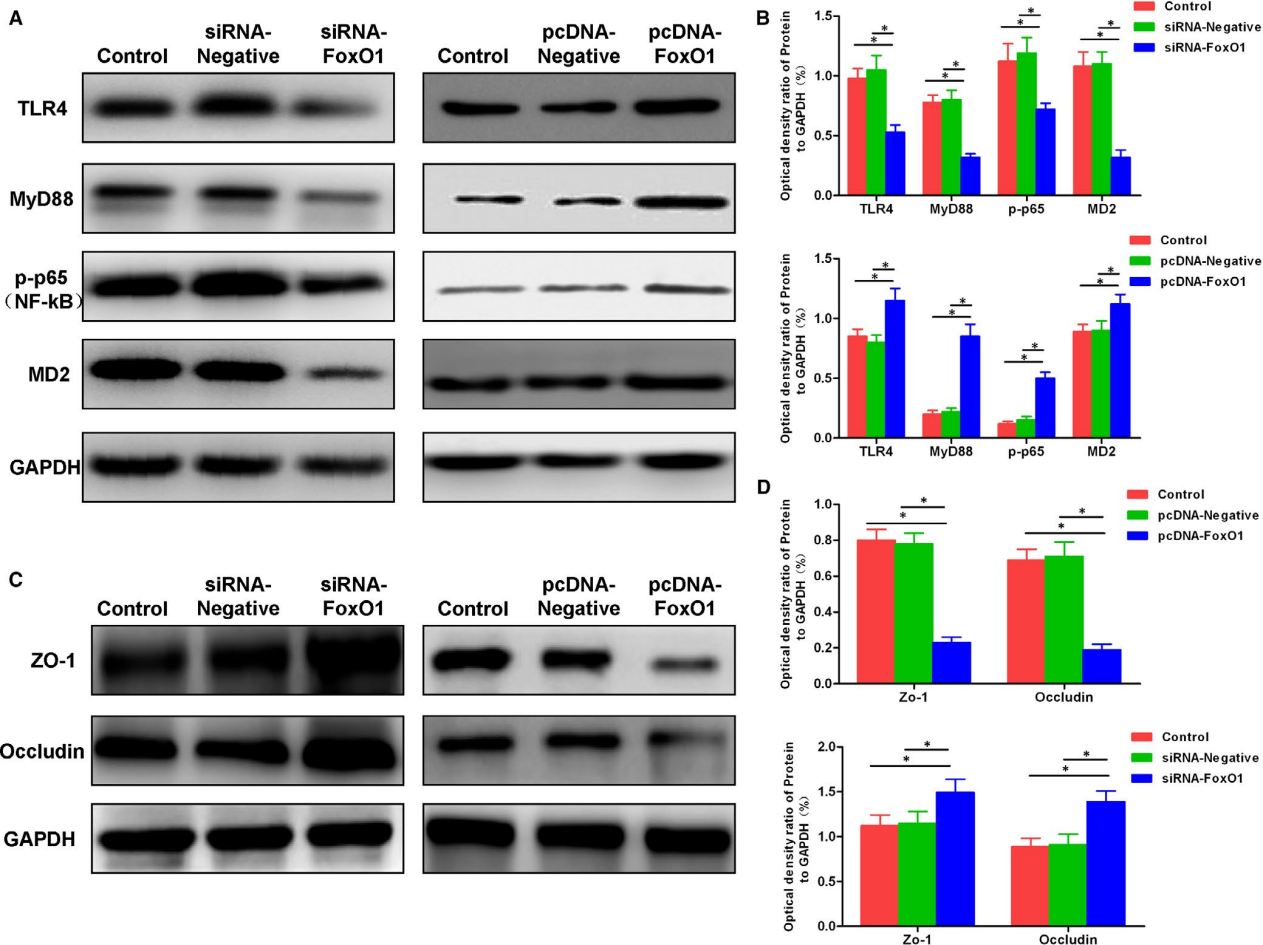
Effects of down‐regulation/overexpression of FoxO1 on inflammatory signalling and tight junction protein expression in inflammatory model. A,B, Expression of TIL4/MyD88/MD2‐NF‐kB signal. The down‐regulation of FoxO1 led to the suppressed TIL4/MyD88/MD2‐NF‐kB signalling pathway, whereas the levels of key proteins TLR4, MyD88 and MD2 were down‐regulated. After FoxO1 overexpression, the levels of key proteins TLR4, MyD88 and MD2 were increased. Comparison between groups, ^*^ *P*<.05. C,D, The expression of tight junction proteins ZO‐1 and occludin. After TNF‐α intervention, the expression levels of tight junction protein were down‐regulated. The down‐regulation of FoxO1 inhibited the down‐regulation of ZO‐1 and occludin proteins, whereas after overexpression of FoxO1, the down‐regulation of ZO‐1 and occludin proteins was more obvious. Comparison between groups, ^*^ *P*<.05

After TNF‐α intervention, the TIL4/MyD88/MD2‐NF‐kB signal was significantly activated, with significantly up‐regulated level of p‐p65, and other key proteins, including TLR4, MyD88 and MD2. After FoxO1 silencing by siRNA, protein levels were down‐regulated, whereas FoxO1 overexpression led to up‐regulated levels of inflammatory signalling proteins, indicating that the expression of FoxO1 was associated with TIL4/MyD88/MD2‐NF‐kB signalling activation. TNF‐α intervention caused the down‐regulation of the tight junction protein level, which was inhibited by FoxO1 silencing by siRNA. On the contrary, FoxO1 overexpression further down‐regulated the tight junction protein levels (shown in Figure 7).

## 4 DISCUSSION

Inflammatory bowel disease is an intestinal inflammatory disease including CD and UC. CD, a chronic granulomatous lesion, can affect the entire colon and terminal ileum,^9^ whereas UC is a chronic, non‐specific inflammation that mainly affects the colonic mucosa and submucosa.^10^, ^11^ The clinical manifestations of IBD include persistent or recurrent diarrhoea, mucopurulent bloody stool accompanied with abdominal pain, with disease course over 4‐6 weeks. The clinical diagnosis of IBD is mainly confirmed by endoscopy. The aetiology of IBD is rather complicated; however, it is relatively clear that the activation of inflammatory signals plays an important role in the pathogenesis and development of IBD. TLRs are a family of receptors involved in immune and inflammatory responses. Recent studies have found that TLR4 plays an important role in IBD.^12^, ^13^ The expression level of TLR4 is down‐regulated in normal human epithelial cells, which is increased in IBD, meanwhile TLR4 can mediate the activation of NF‐kB. In the LPS‐induced inflammatory response, TLR4 can bind to the secretory protein MD2,^14^ further forming a complex with the adaptor protein MyD88 to transfer to the inflammatory response. NF‐kB is inactive in the cytosol without activation.^15^ Once the complex of TLR4, MyD88 and MD2 is formed, IkB can be activated to form an active p‐p65, thereby mediating inflammation response. However, the mechanism of TLR4 signal activation in IBD has not been unveiled.

The FoxO1 transcription factor family plays an important role in cell apoptosis, inflammation and growth cycle.^16^ FoxO1 is one of the important members of the FoxO family and is able to regulate various molecular signals in tissues. Existing studies have shown that after inflammatory damage, the expression of FoxO1 is up‐regulated, which could induce macrophages to produce multiple types of inflammatory factors and aggravate inflammatory damage. In the study of hepatic ischaemia‐reperfusion, FoxO1 is found to be involved in the regulation of NF‐kB,^17^ and the down‐regulation of TLR4 can significantly inhibit hepatic ischaemia‐reperfusion injury.

In consideration of the role of FoxO1 in the inflammatory response, in this study, FoxO1 transgenic mice were used to study its role in IBD. As a result, the expression of FoxO1 in the intestinal tissue was significantly up‐regulated and the TLR4 signal was activated after DSS intervention, indicating that high expression of FoxO1 is associated with the expression of TLR4 signalling. The intestinal mucosal barrier mainly consists of intestinal monolayer columnar epithelial cells and tight junctions. In addition, other intestinal epithelial cells, such as goblet cells and Paneth cells, also participate in maintaining the normal barrier function of the intestinal mucosa by resisting pathogen invasion.^18^, ^19^ Intestinal mucosal barrier injury is considered as a basic pathological change in IBD, accompanied by increased permeability of the intestinal mucosa and changes in villus structures. Among them, tight junction proteins are key proteins to maintain the intestinal mucosal barrier, whereas mucosal damage is mainly accompanied by the loss of tight junction proteins.^20^, ^21^ Similarly, in this study, we also found that overexpression of FoxO1 could lead to further down‐regulation of tight junction proteins, resulting in mucosal damage. At the cellular level, Caco‐2 cells are commonly used to construct intestinal mucosal barrier model. After overexpression of FoxO1, TLR4 signalling was activated and permeability was increased in the inflammatory response model. By contrast, after down‐regulation of FoxO1, the activation level of TLR4 signal was down‐regulated, and the permeability was decreased. These results indicate that FoxO1 can regulate the inflammatory response of IBD by modulating TIL4/MyD88/MD2‐NF‐kB signalling. However, whether FoxO1 predominantly regulates tight junction barrier or inflammatory signalling is uncertain. It is also not clear which signalling comes first during IBD. We speculate that FoxO1, as a transcription factor, may promote the transcription and expression of one protein in TIL4/MyD88/MD2‐NF‐kB signal, thus stimulating the activation of the whole signal. However, the specific protein has not been found, which is also the direction of future research.

To sum up, in this study, we report that the transcription factor FoxO1 can regulate the inflammatory responses and intestinal mucosal injury by modulating TIL4/MyD88/MD2‐NF‐kB signalling. The mechanism of immunology is not involved in this study, which is also the limitation of this study. The immunoregulation and mechanism of FoxO1 in inflammatory bowel disease is the future research content of our group, which is also necessary to further explain the role of FoxO1.

## CONFLICT OF INTEREST

No Competing interests.

## AUTHOR CONTRIBUTIONS

Chenyang Han and Li Guo contributed to Design and operation of the experiment; Yongjia Sheng, Yi Yang and Jin Wang contributed to operation of animal experiment and data processing; Wenyan Li and Xiaohong zhou contributed to collection of clinical samples and detection of inflammatory factors; and Qingcai jiao contributed to the proposal of the subject, the design of the experimental process and the whole process guidance.

## ETHICAL APPROVAL

The study was approved by Ethics Committee.

## CONSENT FOR PUBLICATION

All authors approved the publication of the article.

## Data Availability

The data are available.
