#  

**DOI:** 10.1111/jcmm.17198

**Published:** 2022-03-06

In Chenyang Han et al.^1^, in Figure 3C (DSS‐Foxo1‐Tg) and 3B (Con‐Foxo1‐Tg), the IHC Figure is inserted incorrectly and needs to be corrected. There is an incorrect selection of GAPDH gel in Figure 2F and Figure 3D, incorrect selection of MyD88 in Figure 4C. The correct figures are shown below. The authors confirm that all results and conclusions of this article remain unchanged.

**FIGURE 1:**
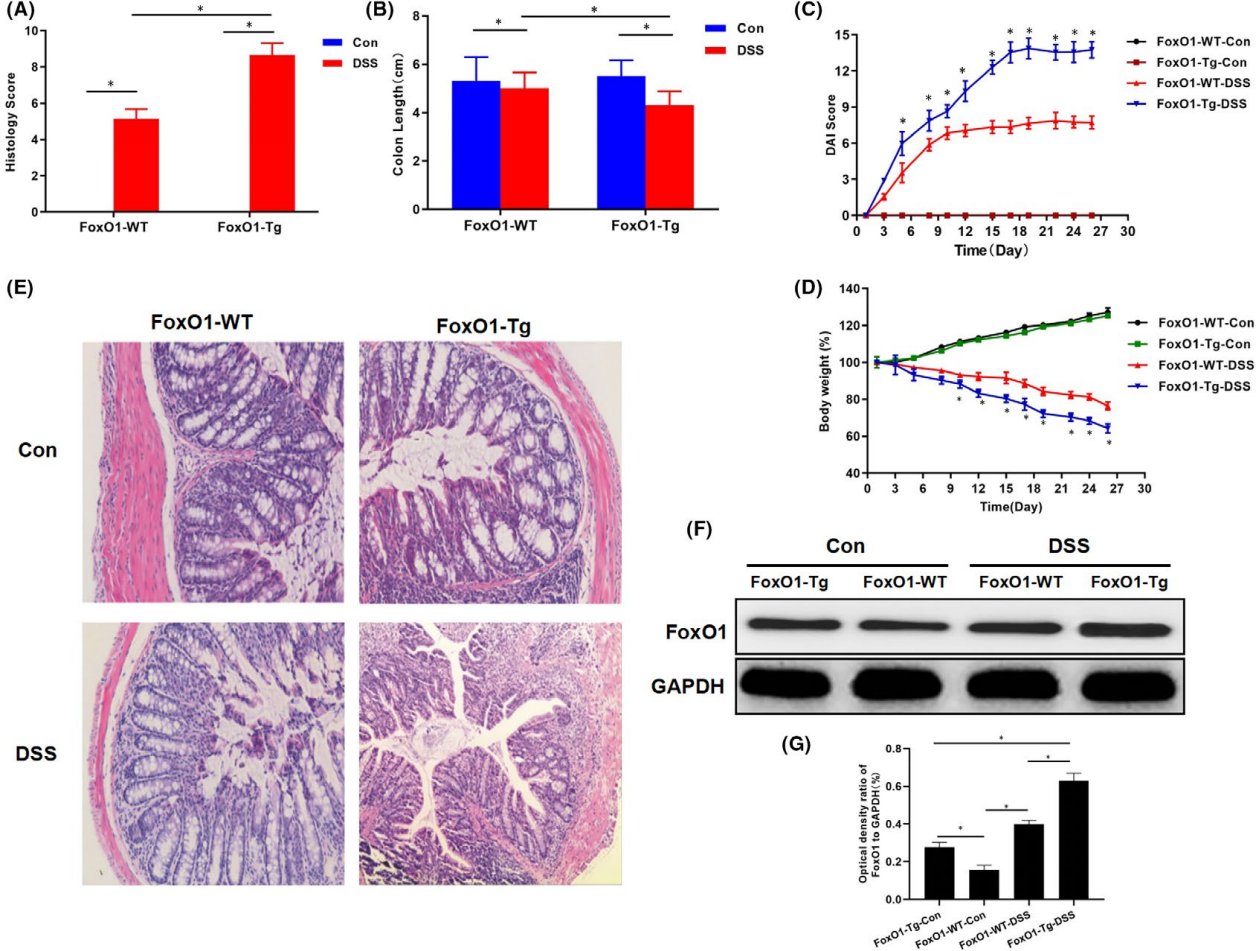
Effects of FoxO1 by evaluating bodyweight, DAI score and pathological condition of mice with chronic colitis. A, The intestinal histopathological scores in mice showed that the pathological scores were 0 in FoxO1‐WT‐Con and FoxO1‐Tg‐Con groups. However, after DSS intervention, the pathological scores were significantly higher in FoxO1‐Tg‐DSS group than those of FoxO1‐WT‐DSS group. Comparison between groups, *P < .05. B, The change in intestinal tissue length in mice: The length of intestinal tissue was significantly lower in FoxO1‐TgDSS group than that in FoxO1‐WT‐DSS group. Comparison between groups, *P < .05. C, DAI scores in mice showed no significant changes in FoxO1‐WT‐Con and FoxO1‐Tg‐Con mice during the experiment, whereas DAI scores were significantly changed in FoxO1‐Tg‐DSS and FoxO1‐WT‐DSS groups. Comparison with FoxO1‐WT‐DSS group at the same time‐point, *P < .05. D, Bodyweight changes of mice: The bodyweight was gradually increased in FoxO1‐WT‐Con and FoxO1‐Tg‐Con mice under normal feeding, without significant difference. However, the bodyweight was decreased in FoxO1‐Tg‐DSS and FoxO1‐WT‐DSS groups. Comparison with the FoxO1‐WT‐DSS group at the same time‐point, *P < .05. E, HE staining showed that the intestinal mucosa epithelium was intact, and the intestinal gland composed of lamina propria and mucosa muscle layer was arranged regularly in FoxO1‐WT‐Con group under light microscope. However, the colonic mucosa was defective, with decreased goblet cells and destructive or even disappeared gland, and large number of lymphocytes were infiltrated in the submucosa and even the muscle layer in FoxO1‐WT‐DSS group, which was more severe in FoxO1‐Tg‐DSS group than FoxO1‐WT‐DSS group. F,G, The expression of FoxO1 in mouse intestinal tissue: The expression of FoxO1 was increased in FoxO1‐WT‐DSS and FoxO1‐Tg‐DSS groups after DSS intervention, and the expression of FoxO1 was significantly higher in FoxO1‐Tg‐DSS group than that in FoxO1‐WT‐DSS group. Comparison between groups, *P < .05

**FIGURE 2:**
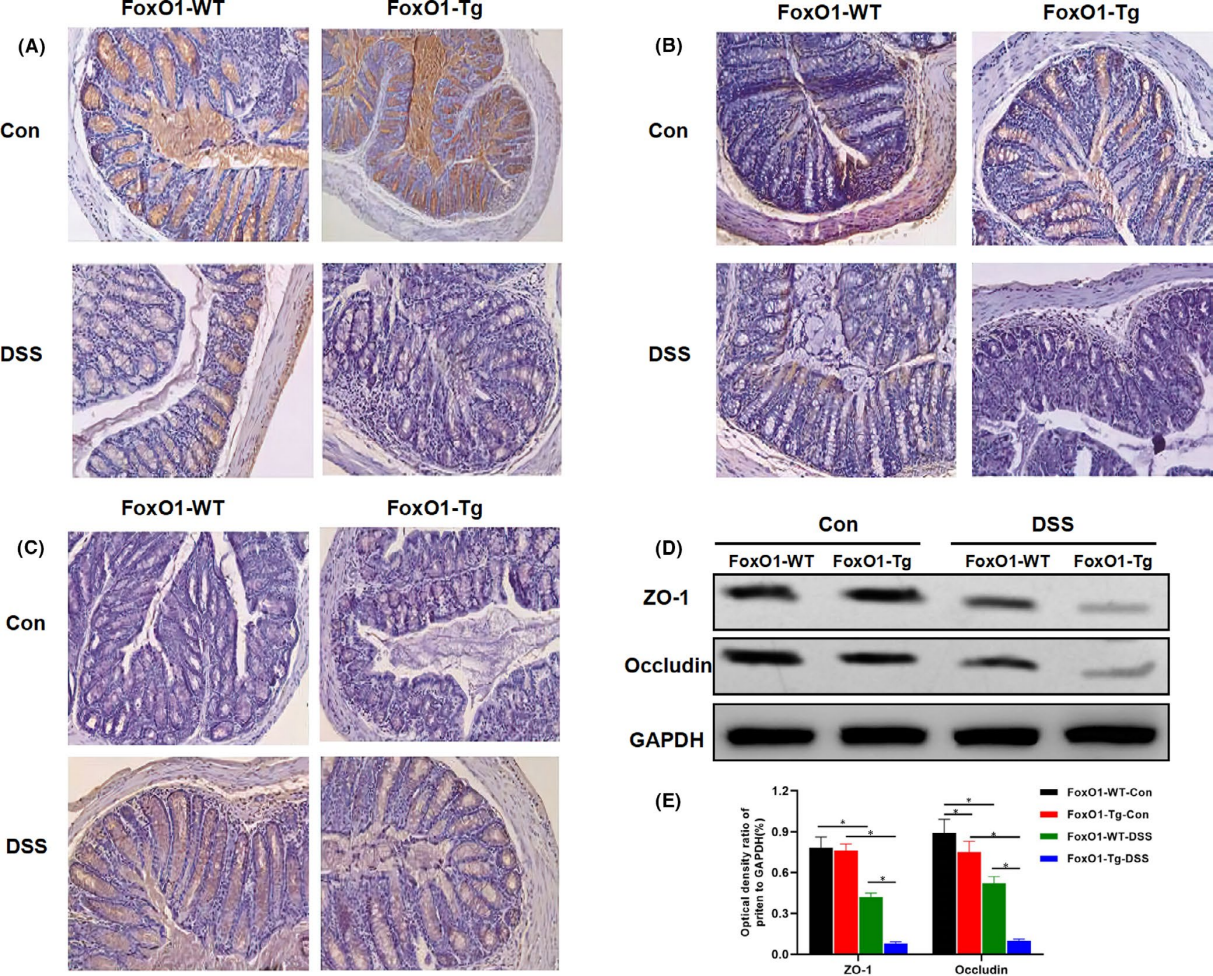
Effects of FoxO1 on the expression of tight junction protein in mice with chronic colitis. A, Expression of ZO‐1 protein in mouse intestinal tissue by IHC: ZO‐1 protein was strongly expressed in intestinal tissues of FoxO1‐WT‐Con and FoxO1‐Tg‐Con groups, which was significantly down‐regulated in FoxO1‐WT‐DSS and FoxO1‐Tg‐DSS groups. Meanwhile, the expression of ZO‐1 was significantly lower in FoxO1‐Tg‐DSS group than that of FoxO1‐WT‐DSS group. B, Expression of occludin protein in mouse intestinal tissue by IHC: Occludin protein was strongly expressed in intestinal tissues of FoxO1‐WT‐Con and FoxO1‐Tg‐Con groups, which was significantly down‐regulated in FoxO1‐WT‐DSS and FoxO1‐Tg‐DSS groups. Meanwhile, the expression of occludin was significantly lower in FoxO1‐Tg‐DSS group than that of FoxO1‐WT‐DSS group. C, Expression of FoxO1 protein in mouse intestinal tissue by IHC: The expression levels of FoxO1 were significantly increased in FoxO1‐WT‐DSS and FoxO1‐Tg‐DSS groups after DSS intervention, whereas the expression levels of FoxO1 were significantly higher in FoxO1‐Tg‐DSS group than those in FoxO1‐WT‐DSS group. D,E, The protein expression of ZO‐1 and occludin in intestinal tissue by Western blot. After DSS intervention, the expression levels of ZO‐1 and occludin were down‐regulated, whereas the expression levels of ZO‐1 and occludin were significantly lower in FoxO1‐Tg‐DSS than those in FoxO1‐WT‐DSS group. Comparison between groups, *P < .05

**FIGURE 3:**
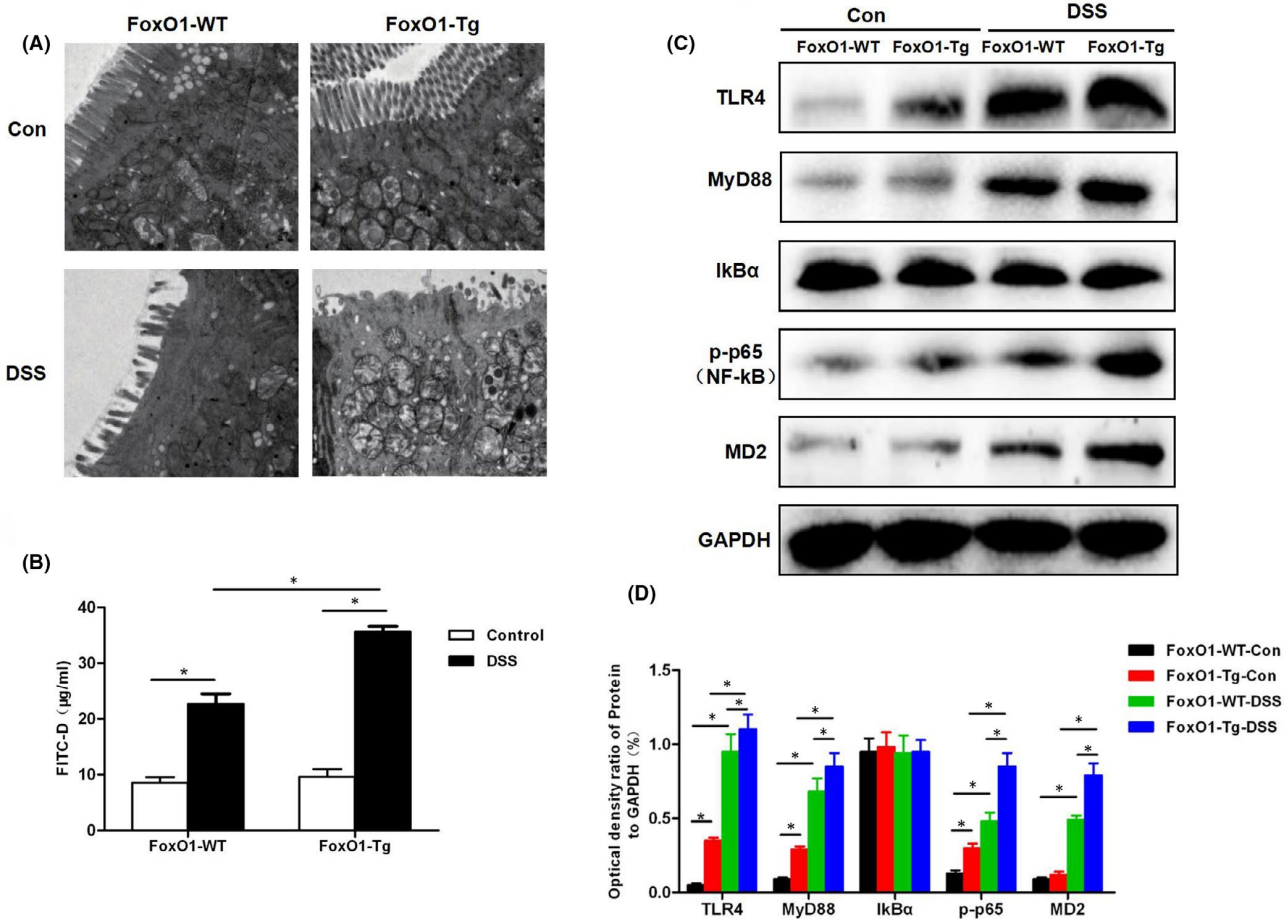
Effects of FoxO1 on intestinal mucosal villus structure, permeability and expression of inflammatory signals in mice with chronic colitis. A, Changes in the intestinal mucosal microvillus structure of mice. The intestinal mucosal microvillus was neatly arranged in FoxO1‐WT‐Con and FoxO1‐Tg‐Con mice, with clear structure. The intestinal mucosa microvillus structure was significantly changed in FoxO1‐WT‐DSS and FoxO1‐Tg‐DSS groups, with obvious loss of microvillus structure. In addition, the lesion degree was significantly more severe in FoxO1‐Tg‐DSS group than that in FoxO1‐WT‐DSS group. B, FITC‐D permeability assay revealed that the permeability was significantly higher in FoxO1‐WT‐DSS and FoxO1‐Tg‐DSS groups than that in Con group. Comparison between groups *P < .05. C,D, Western blot assay for TIL4/MyD88/MD2‐NF‐kB signalling. The activation levels of TIL4/MyD88/MD2‐NF‐kB signalling were lower in FoxO1‐WTCon and FoxO1‐Tg‐Con groups. After DSS intervention, the levels of key proteins, TLR4, MyD88 and MD2 were up‐regulated. Comparison between groups, *P < .05
